# Biodegradable Polycaprolactone Fibers with Silica Aerogel and Nanosilver Particles Produce a Coagulation Effect

**DOI:** 10.3390/polym15092022

**Published:** 2023-04-24

**Authors:** Büşra Şengel Ayvazoğlu, Muhammet Ceylan, Aybüke A. Isbir Turan, Elif Burcu Yılmaz

**Affiliations:** 1 Institute of Forensic Sciences, Turkish National Police Academy, 06834 Ankara, Turkey; 2 Graduate School of Natural and Applied Sciences, Istanbul Ticaret University, 34840 Istanbul, Turkey

**Keywords:** aerogel, antimicrobial effect, coagulation effect, electrospinning, nano-silver, fiber, polycaprolactone

## Abstract

Poly-ε-caprolactone (PCL) is a biodegradable aliphatic polyester that can be used in the field of biomaterials. Electrospinning is the name given to the process of producing micro and nanoscale fibers using electrostatically charged polymeric solutions under certain conditions. Almost all synthetic and naturally occurring polymers can undergo electrospinning using suitable solvents or mixtures prepared in certain proportions. In this study, silica aerogels were obtained by the sol-gel method. PCL-silica aerogel fibers were synthesized by adding 0.5, 1, 2, and 4% ratios in the PCL solution. Blood contact analysis was performed on the produced fibers with UV-VIS. According to the results obtained, 0.5, 1, 2, and 4% nano-silver were added to the fiber-containing 4% aerogel. Then, SEM-EDS and FTIR analyses were performed on all fibers produced. Antimicrobial tests were performed on fibers containing nano-silver. As a result, high-performance blood coagulation fibers were developed using PCL with aerogel, and an antimicrobial effect was achieved with nano-silver particles. It is thought that the designed surface will be preferred in wound dressing and biomaterial in tissue engineering, as it provides a high amount of cell adhesion with a small amount of blood and contains antimicrobial properties.

## 1. Introduction

Silica aerogels have a low-density mesoporous solid structure that is widely used in applications in engineering and science [1]. In recent years, aerogels have had the potential to be used in the medical field, such as tissue engineering substrates [2], drug delivery systems [3], artificial bone grafts [4], and artificial heart valves [5]. The revival of sol-gel technology owes to the production of silica aerogel for the storage of rocket propellants. Sol-gel nanomaterial production is one of the liquid-phase synthesis methods. The sol-gel method enables nanomaterials to be produced in small sizes at low temperatures and economic conditions. The sol-gel process has five basic steps: hydrolysis, polycondensation, aging, drying, and high temperature [6,7].

The petroleum-based biodegradable polyester Poly(ε-caprolactone) (PCL) was one of the first polymers synthesized by the Carothers group in the early 1930s [8]. PCL is a linear, aliphatic semi-crystalline biodegradable thermoplastic polyester composed of repeating hexanoate units. The production of PCL is relatively more economical than its equivalent aliphatic polyesters, so it is widely used in developing biomedical devices [9]. PCL is relatively hydrophobic. PCL has a low melting point range, good chemical and oil resistance, lower viscosity, and easy-to-process thermal properties [10,11,12,13].

Nano-silver is a tiny particle that can be synthesized by physical, chemical, or biological methods [14]. Nano-silver production occurs through nucleation and growth of various inorganic phases in the atmosphere, hydrosphere, and even in the lithosphere (melts) with mechanical and thermal processes [15,16,17]. In addition, nano-silver particles (AgNPs) interact with abiotic and biotic components through various interconnected processes [18]. AgNPs are one of the essential studied types of nanomaterials. AgNPs have unique sensing, catalytic, optical, and antimicrobial properties and are used as efficient probes for detecting various biomolecules and monitoring biotransformations [19,20,21].

Similarly, nano-gold particles (AuNPs) are used in chemical and biological imaging, diagnostics, cancer therapy, sensors, and catalysis [22,23]. However, AgNPs are preferred in smart drug delivery systems with their properties such as controllable delivery and release in response to various stimuli such as enzyme reaction, magnetic force, light, pH, and temperature [24]. Nanoparticles such as gold, silver, titanium, copper, and iron exhibit antibacterial effects with their structure, shape, size, and surface modification properties. While AuNPs show antibacterial properties at 2 nm size, AgNPs show higher antibacterial properties with triangular nanoplates when compared with other shapes [25]. 

Electrospinning is a method that enables the production of fibers ranging from nanometers to micrometers with electrostatic force [26]. Electrospinning is based on the principle that the liquid placed in the syringe is exposed to a high electric field with the help of a power source. When the liquid overcomes the surface tension and creates a jet (Taylor jet), nano-micro-sized fiber structures are produced on the collector plate. Electrospinning is a very economical and efficient method to produce nanofibers from many different polymer types [27]. Fibers produced by the electrospinning method have a high surface area/volume value and extremely small pores. Therefore, it has applications in many fields such as nanocatalysis, tissue engineering, protective textiles, filtration, biomedical, pharmaceutical, optical electronics, health, biotechnology, safety, and environmental engineering [28,29,30].

Nanofibers are fibers obtained by various methods, including self-assembly, template synthesis, electro-spinning, and phase separation. In addition, nanofibers can be prepared using various polymers such as cellulose, collagen, keratin, gelatin, silk fibroin, poly (lactic-co-glycolic acid) (PLGA), poly (lactic acid) (PLA), poly (ethylene-co-vinyl acetate) (PEVA), polysaccharides, and PCL. Nanofibers are considered important by many researchers. Nanofibers offer qualities such as having high surface-to-volume ratios, being lightweight, and having small diameters and controllable pore structures [31,32,33]. Among the electrospinning polymers, PCL is well known to form nanofibers in a wide range of compositions and products [34]. Anand et al., synthesized PCL nanofibers treated with Kurdlan sulfate and heparin. Fewer adherent platelets were observed on the PCL-CURS and PCL-HEP surfaces compared with the normal PCL surface [35]. Victoria and Ketul preferred PCL nanofibers treated with collagen and a different nanosurface called nanowire. Control (PCL nanofiber), PCL nanowire, collagen-treated control (PCL nanofiber), and collagen-treated nanowire surfaces were synthesized. Platelet–leukocyte interaction increased on control PCL surfaces and PCL surfaces treated with collagen. In addition, no toxic effects were observed in blood interaction with nanosurfaces [36]. Q. Shi et al., produced nanofibers by using the single spinneret electrospinning method from mixtures of poly (N-isopropyl acrylamide) (PNIPAAm), PCL, and nattokinase (NK) solution. Platelets tend to adhere to the hydrophobic surface in nature. Platelets adhere to the nanofiber surface due to the hydrophobic surface of PCL/PNIPAAm nanofibers at 37 °C, and PCL/PNIPAAm/NK nanofibers resist platelet adhesion at 37 °C due to the presence of NK. [37]. Jun-Yong et al., synthesized PCL nanofibers containing calcium carbonate (CaCO_3_) and β-chitosan. The highest blood coagulation rate was found in polycaprolactone nanofibers with CaCO_3_ content coated with β-chitosan. In addition, similar results were obtained in nanofibers tested in open wounds, and the fastest clotting was observed in polycaprolactone nanofibers with CaCO_3_ content coated with β-chitosan [38].

Natural polysaccharides are very important natural macromolecules and have great potential in wound treatment because of their excellent biological properties. Several common natural polysaccharides such as chitosan, starch, alginate, and hyaluronic acid are used in wound healing by electrospinning [39]. Ho et al. fabricated an electrospun polycaprolactone (EsPCL) membrane coated with various densities of chitosan oligomers (COS) for application as a bioactive wound dressing. COS altered the thickness of the polymeric membrane, which resulted in stronger antibacterial activities. In terms of hemostasis, the increase in COS density on the membrane accelerated blood coagulation and also affected the reepithelization and wound healing in mice. Thus, the membrane and particularly chitosan oligomers were shown to have the potential for wound healing [40]. Waghmare et al. fabricated starch-based nanofibers scaffolds by electrospinning starch-based nanofibers tested cellular assays with L929 mouse fibroblast cells. It promoted cellular proliferation without exhibiting any toxic effect on the cells and demonstrated potential for wound healing applications [41].

The aim of this study focused on the manufacture of a wound dressing with biocompatible materials. It used three components: PCL, aerogel, and nano-silver. Firstly, PCL was chosen because it is a biodegradable material and has limited properties for biological application due to its hydrophobicity. Second, aerogel is a natural safety material that plays an important role in accelerating blood coagulation properties. In addition, aerogel is used for surface property modification to permeate blood easily into fiber and increase the fiber’s contact area. Third, nanosilver is used for antimicrobial properties. In addition, electrospinning fibers were produced using these three components to increase the surface area to come into contact with blood. PCL fiber mat was used as a substrate and aerogel was added to the fiber solution. Then, the nanosilver solution was coated on the fiber surface. Blood coagulation characteristics of PCL and PCL/aerogel fibers were compared. It was found that PCL/aerogel fibers with nano silver exhibited a higher blood coagulation rate than the cotton surface. Therefore, PCL/aerogel fibers with nano silver are expected to be useful for wound dressing.

## 2. Materials and Methods

### 2.1. Materials

Sodium silicate solution (Na_2_SiO_3_), hydrochloric acid (MW: 36.46)(HCl), methanol (MeOH), n-hexane (CH_3_(CH_2_)_4_CH_3_)(MW:86.18), silane (SiH_4_), polycaprolactone (6-caprolactone polymer, 2-oxepanone homopolymer) (C_6_H_10_O_2_)n (M.W.:80,000), chloroform (CHCl_3_) (MW: 119.38 g/mol), and methanol (CH_3_OH) (MW: 32.04 g/mol) were obtained from Sigma Aldrich, in Turkey. In addition, nano-silver powder (5 nm) was obtained from Nanokar, in Turkey.

### 2.2. Synthesis of Silica Aerogel

**Figure 1:**
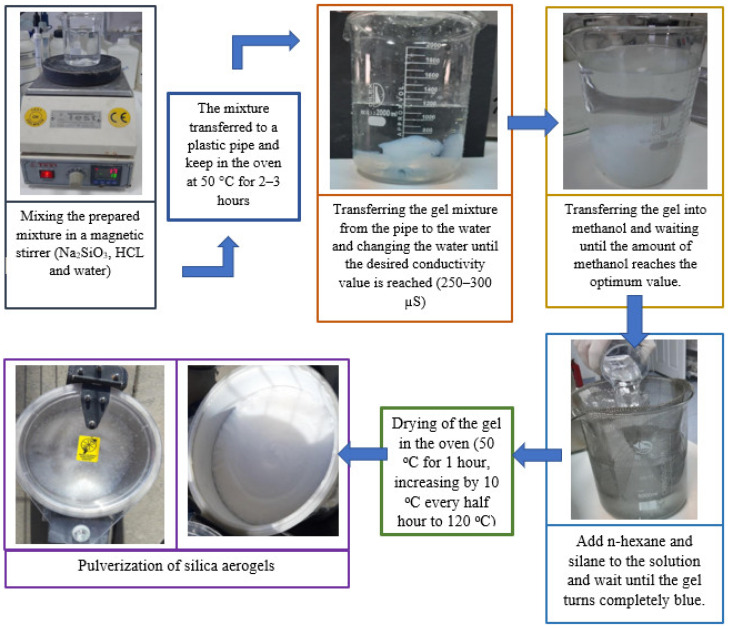
The production steps of silica aerogel.

An amount of 20 mL of Na_2_SiO_3_, 10 mL of HCl, and 150 mL of water were put into a beaker and mixed for a specific time in a Daihan MSH-20D magnetic stirrer. The prepared mixture was thoroughly mixed and transferred to a plastic pipe. Afterward, the pipe was kept in an oven at 50 °C for approximately 2–3 h. Then, the gelling mixture was removed from the pipe and left in the water in a beaker. After a certain period, a conductivity measurement of the gel was performed. The water was changed until the desired measurement value is reached. The final conductivity value was measured between 250 and 300 µS. The desired conductivity levels were achieved, and the gel was deposited in methanol. The resulting gel was kept in a Daihan WAS-D06H ultrasonic bath for approximately 8–10 h. After waiting in methanol for a particular time, the first alcohol measurement was made. The measured value indicates the amount of methanol in the gel. As a result of the first measurement, the amount of methanol in the gel was determined to be 94%. This gel was kept in a methanol solution for approximately one day. After the first measurement, alcohol measurement was made at specific intervals. If there is no significant change in the measurement value, it means that the amount of methanol in the gel has reached the optimum value. The gel, which reaches the optimum value, completes the methanol process and switches to the n-hexane stage. In the n-hexane solution stage, a silane chemical was added to the solution so that the resulting gel turns blue. It was waited by adding n-hexane and silane to the top of the gel. The waiting time for the mixture is uncertain. The n-hexane process is complete when the gel turns completely blue. The final stage is the drying stage. The gels were filtered and taken into a baking dish. First, it was kept in the Protherm oven at 50 °C for about 1 h. Then, the temperature of the oven was increased by 10 °C every half hour and increased by 120 °C. The purpose of the furnace step is to evaporate the n-hexane and silane contained in the gel and to allow the gel to reach the aerogel structure. The resulting silica aerogels are in the form of small particles. Silica particles were pulverized with the help of a grinder. The production steps of silica aerogel are shown in Figure 1 [42].

### 2.3. Fabrication of Silica Aerogel/PCL Fibers

**Figure 2:**
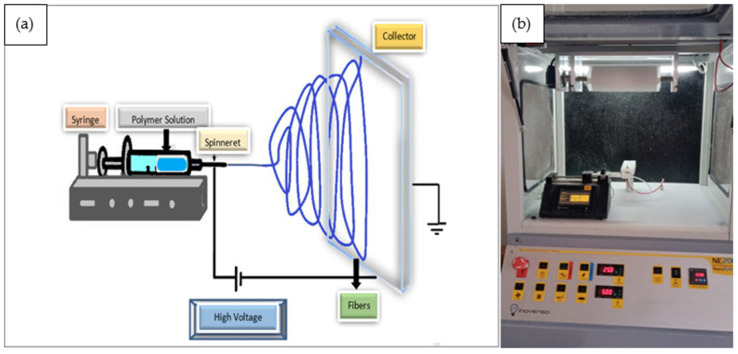
The systematic production of fiber with electrospinning methods (**a**) and the photo of the electrospinning machine (**b**).

In the preparation of fibers, methanol–chloroform (MET-CL) solvent groups were used at a weight ratio of 75:25. PCL was dissolved in solvent groups at a weight ratio of 85:15, and the polymeric solution was obtained. Silica aerogel was added to the polymeric solution at the weight rates of 0.5, 1, 2, and 4%. This solution was kept in a DAIHAN MSH-20D magnetic stirrer at 300 rpm at 50 °C until homogeneously mixed. The polymeric solution was transferred into a 10 mL plastic syringe and placed in the NE 200 NanoSpinner electrospinning device, Inovenso. First, a high-voltage electric field was formed between the spinning nozzle and the collector using a high-voltage power supply. The electrostatic force promotes the stretching and formation of fibers by the droplet. The flow of polymer solution from the syringe was controlled by a programmable syringe pump. The fibers were sprayed at ~25 KV with a flow rate of 0.750 mL/hr. The solvent in the spinning fluid between the spinning nozzle and the collector was continuously volatilized. The distance between the cylinder collector and the nozzle was 30 cm (Figure 2). 

### 2.4. Impregnation of AgNPs into Fibers

AgNPs were added at the weight ratio of 0.5, 1, 2, and 4% compared with the amount of polycaprolactone in the dipping solution. AgNPs were dissolved in 20 mL of ethanol. The nanoparticle solution was added to each of the fibers. Then, the fibers were dried in an oven at 50 °C for approximately one hour.

### 2.5. Whole-Blood Coagulation Assessment

For assessment of blood coagulation by fibers, fiber mat samples were cut to 1.5 cm^2^ and attached to glass slides. An amount of 0.1 and 0.05 mL of blood were dropped into each sample, respectively. Samples were then dried at room temperature for 20 and 40 min. To remove non-clotted blood, glass slides containing fiber mats were placed in a 50 mL distilled water bath with magnetic stirring at 250 rpm for 15 s. A sample of 1.5 mL of solution was taken from the 2 mL bottle and was centrifuged at 1000 rpm for 1 min. An ultraviolet-visible (UV-VIS) spectrophotometer was used to measure the optical density of the centrifuged solution.

### 2.6. Zone Inhibition Antimicrobial Activity Test

4% PCL/aerogel fibers of impregnated 0.5, 1, 2, and 4% AgNPs were analyzed with Escherichia coli (*E. coli)* and Staphylococcus aureus (*S. aureus)* bacteria. A Luria–Bertani (LB) medium consisting of yeast extract, peptone (casein), sodium chloride (NaCl), and agar was prepared and poured into plates. *E. coli* and *S. aureus* bacteria were inoculated on an LB medium. The density of the suspensions was adjusted to equal the McFarland standard corresponding to approximately 1–2 × 10^8^ CFU/mL of *E. coli* and *S. aureus*. The fibers were cut 8 mm in diameter and were sterilized under ultraviolet light. The placed fibers in plates were incubated for 18–24 h at 37 °C. The zones of inhibition were read.

## 3. Results and Discussion

### 3.1. Characterization of Silica Aerogel

**Figure 3:**
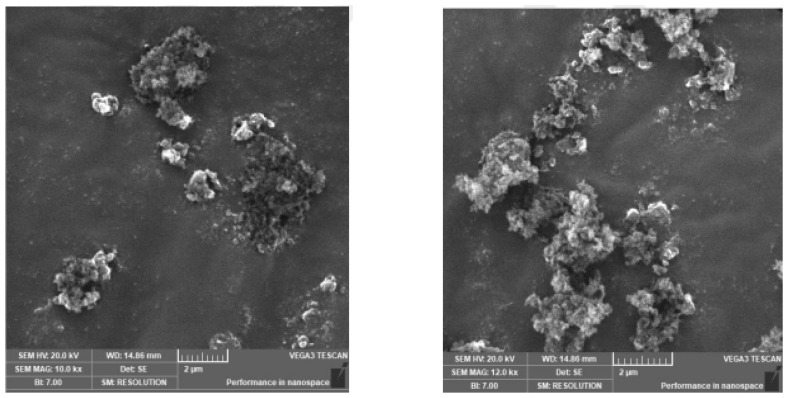
SEM images of silica aerogel were obtained at zoom in 10.0 and 12.0 kx.

**Figure 4:**
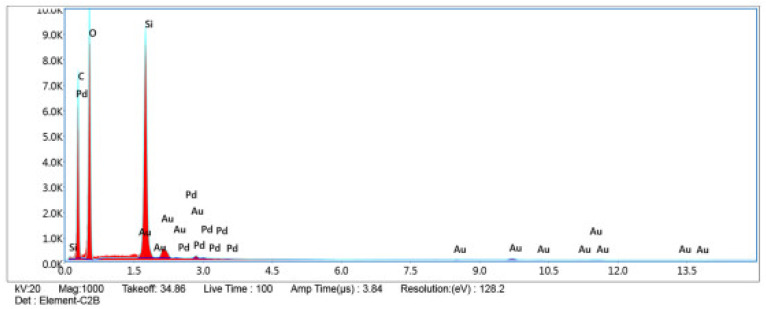
Result of SEM-EDS analysis of silica aerogel.

The morphological structure of the silica aerogel produced by the sol-gel method was investigated by scanning electron microscopy (SEM). Figure 3 shows SEM images of the silica aerogel. Elemental analysis of silica aerogel was performed with SEM-EDS. Figure 4 shows the result of the SEM-EDS analysis. As a result of EDS analysis, the highest silicon (Si) element was found in the content of silica aerogel.

### 3.2. Fiber Morphology and Aerogel Content

**Figure 5:**
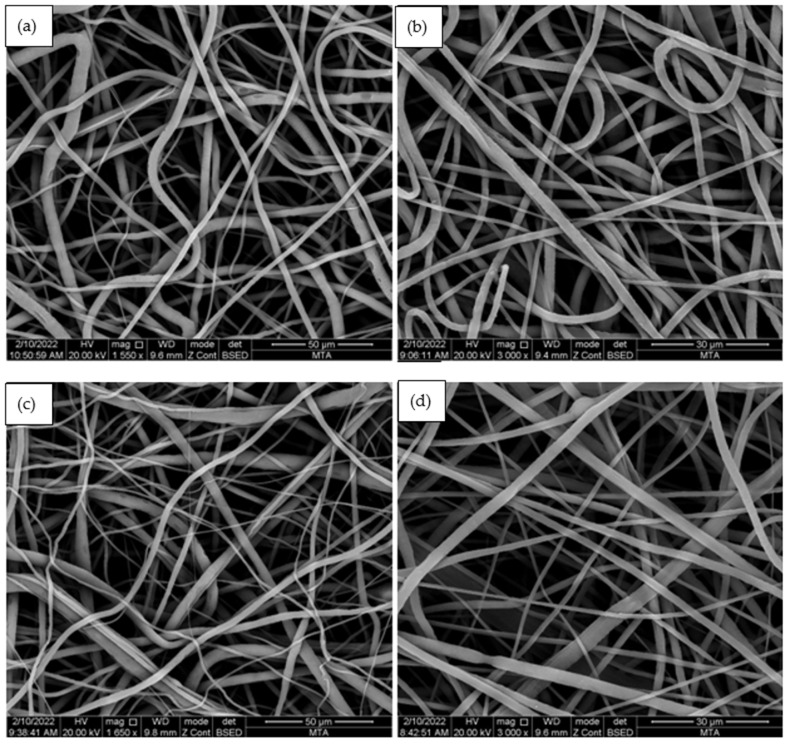

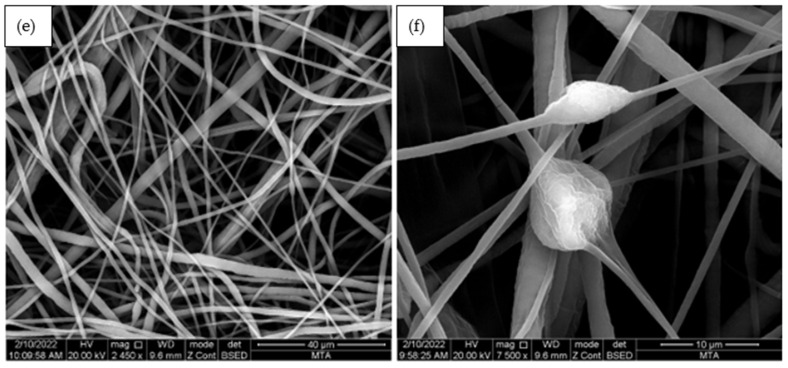
Scanning electron microscope-energy-dispersive X-ray spectroscopy images of (**a**) the PCL fiber, the (**b**) 0.5, (**c**) 1, (**d**) 2, and (**e**) 4% PCL/aerogel fiber, and (**f**) silica beads in 4% PCL/aerogel fiber.

**Figure 6:**
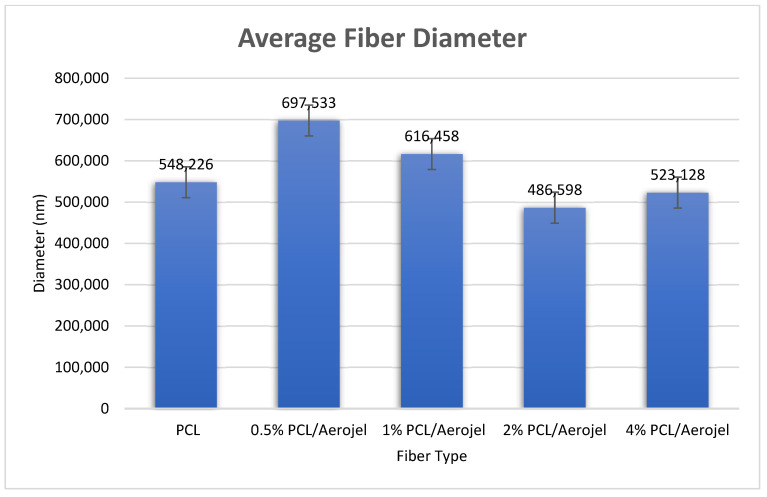
The average fiber diameter of PCL fibers with different concentrations of aerogel.

Using PCL and aerogel, fibers were fabricated via electrospinning. PCL and PCL/aerogel fiber morphologies were measured by SEM-EDS (Figure 5). The PCL and PCL/aerogel fiber diameters were approximately 400–700 nm. The PCL fiber is an ultrafine fiber without beads. In contrast, the 4% PCL/aerogel fiber includes Si beads. A higher magnification Si beads image is shown in Figure 5f. Carbon (C) and oxygen (O) elements were detected in the PCL fiber. C and O elements originating from polycaprolactone and silica elements originating from aerogel were detected in 0.5, 1, 2, and 4% PCL/aerogel fibers. In addition, It was confirmed that the bead structure detected in 4% PCL/aerogel fiber originated from silica with a high percentage of silicon (Table 1). The obtained SEM images were transferred to the ImageJ program. Approximately 10 strands were selected for each fiber with the ImageJ program. Then, the average fiber diameter graph was obtained by taking the standard deviation of the measured dimensions of these strands (Figure 6). 

**Figure 7:**
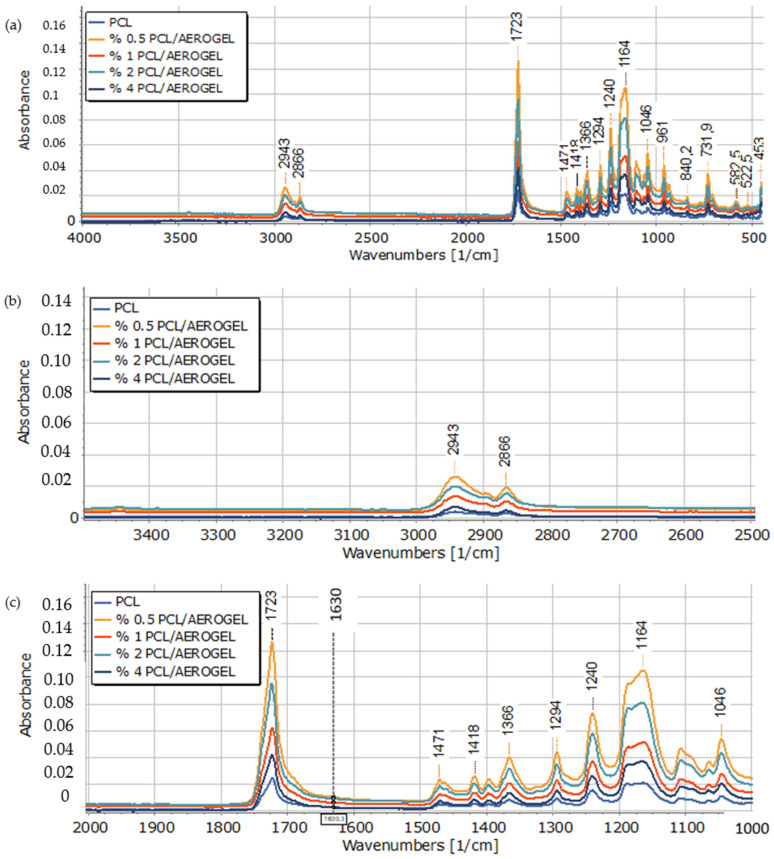
Results of FT-IR spectrum. (**a**) Wide range of FT-IR spectrum (500–4000 cm^−1^). (**b**) The narrow range of FT-IR spectrum (3500–2500 cm^−1^). (**c**) The narrow range of FT-IR spectrum (2000–1000 cm^−1^). The dark dashed line indicates an absorbance peak at 1630 cm^−1^.

The functional properties of the produced fibers were measured with the help of Fourier Transform Infrared Spectroscopy (FT-IR). The spectrum of all fibers showed absorption peaks at about 2950–2940 cm^−1^ and 2870–2860 cm^−1^. The strong band at 3000–2850 cm^−1^ corresponded to the aliphatic C-H stretching of polycaprolactone. The peak at 2950–2940 cm^−1^ was attributed to asymmetric CH_2_ stretching. The peak at 2870–2860 cm^−1^ is due to the symmetrical CH_2_ bond stretch [43]. Hydrophilicity and hydrophobicity were determined according to the FTIR spectrum ranges of the silica aerogel by Wang et al. It was shown that if the silica aerogel is hydrophilic, a peak should be observed at approximately 3400 cm^−1^, which is thought to originate from free or adsorbed water. In addition, this should be accompanied by a spectrum at 1630 cm^−1^, which is the characteristic point of the Si-OH bond responsible for hydrophilicity. It has been stated that if the silica aerogel is hydrophobic, there should be a spectrum at 2950 cm^−1^ corresponding to the C–H bond, and the spectral density at 3400 cm^−1^ and 1630 cm^−1^ should decrease compared to those stated for hydrophilic silica aerogels [44]. 0.5% PCL/Aerogel, 1% PCL/Aerogel, 2% PCL/Aerogel, and 4% PCL/Aerogel fibers showed absorption peaks at a spectrum in the range of 2950–2940 cm^−1^. 0.5% PCL/Aerogel, 1% PCL/Aerogel, 2% PCL/Aerogel, and 4% PCL/Aerogel fibers have no absorption peaks at the spectrum in the range of 1630 cm^−1^, which is the characteristic peak of the Si-OH bond responsible for hydrophilicity. All results are shown in Figure 7.

### 3.3. Whole-Blood Coagulation Assessment

**Figure 8:**
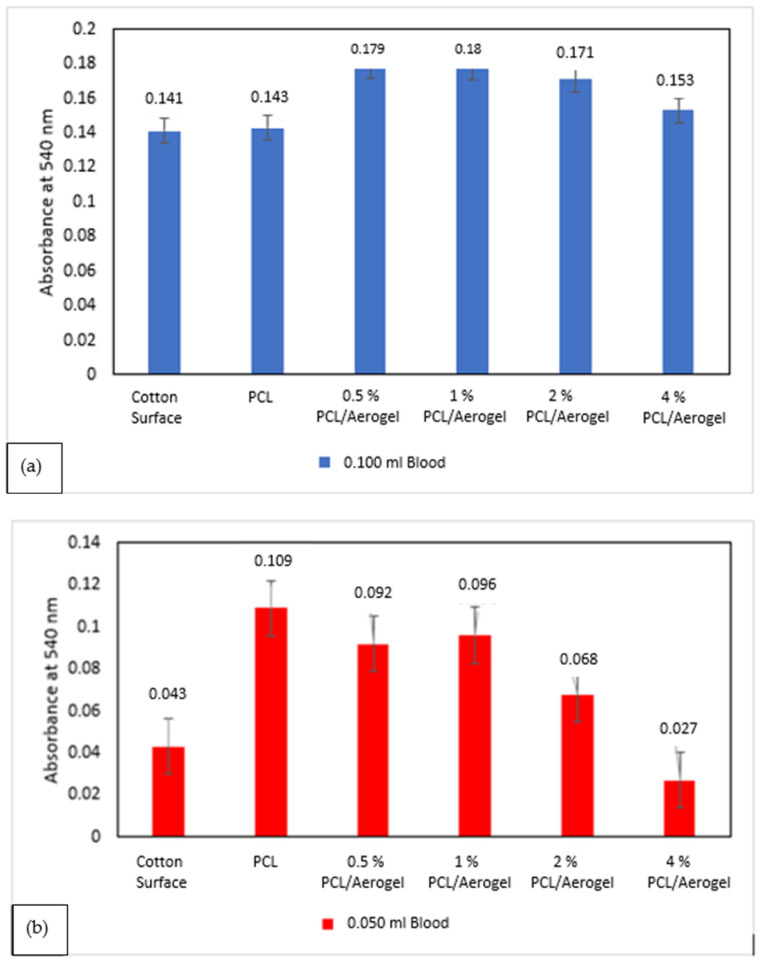

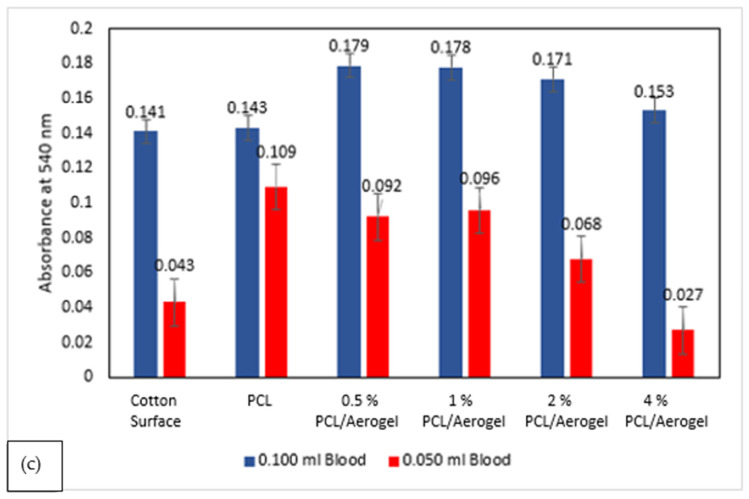
Results of whole-blood coagulation experiment using human blood on glass slides. Fiber mats were analyzed with (**a**) 0.100 or (**b**) 0.050 mL blood. All other experimental conditions were the same. (**c**) The results are shown in the common graph.

The clotting abilities of cotton, PCL, and PCL/aerogel fibers on glass slides were assessed, and the measured absorbance of the non-clotted blood was at 540 nm (Figure 8). A lower absorbance means it reflects less hemoglobin in the sample. Therefore, it shows a higher coagulation rate. The absorbances of cotton, PCL, and PCL/aerogel fibers were assessed with amounts of 0.100 and 0.050 mL of blood (Figure 7a,b). The absorbance of the cotton surface was almost the same as that of the PCL fiber alone in 0.100 mL of blood. However, PCL fibers with aerogel exhibited higher absorbances than cotton in 0.100 mL of blood. The absorbance of the cotton surface was lower than PCL fiber alone in 0.050 mL of blood. However, PCL fiber with the highest aerogel exhibited higher absorbances than cotton in 0.050 mL of blood. The fact that the PCL/aerogel fiber exhibited a higher clotting rate than the PCL fiber confirms that aerogel affects the blood coagulation process. This showed the fact that aerogel increases red blood cell coagulation through hydrophobic interaction. In comparing the samples, we found that the lowest amount of blood consistently exhibited higher absorbances (Figure 8c). This is because nano scales provide the attachment of the lowest amount of blood to the surface of the fibers.

**Figure 9:**
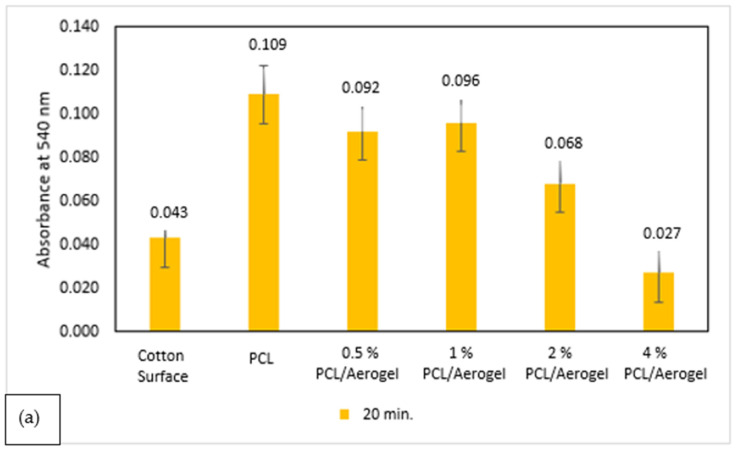

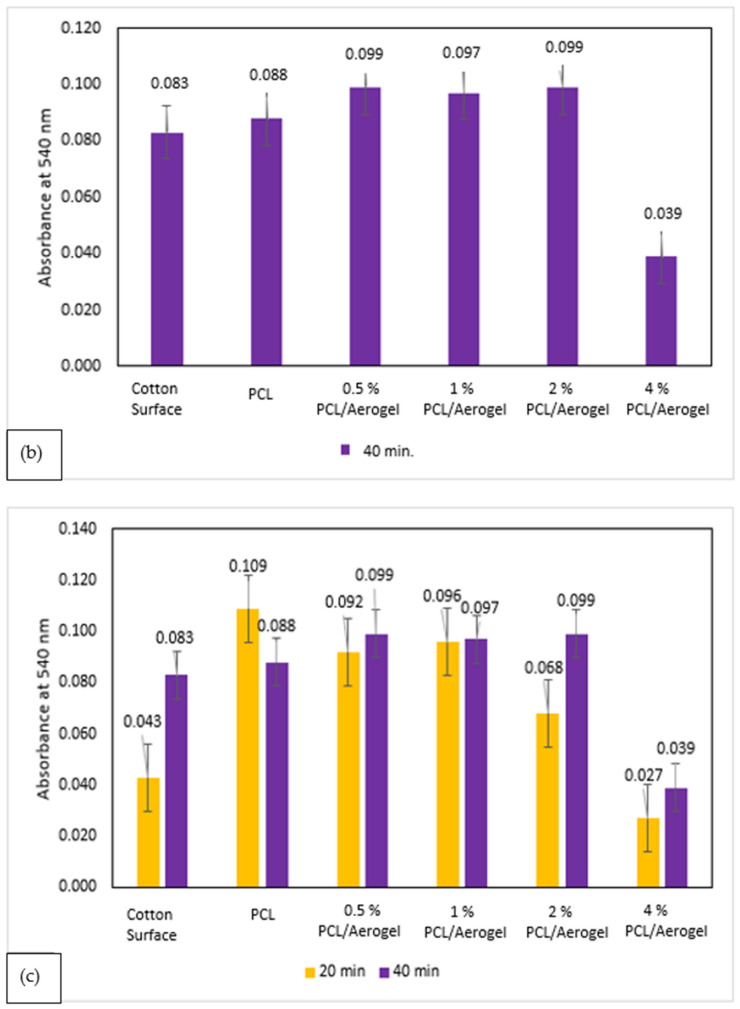
Results of whole-blood coagulation experiment using human blood on glass slides. Fiber mats were analyzed with 0.050 mL blood at (**a**) 20 or (**b**) 40 min. All other experimental conditions are the same. (**c**) The results are shown in the common graph.

The absorbances of cotton, PCL, and PCL/aerogel fibers were assessed with coagulation times of 20 min and 40 min in the amount of 0.050 mL blood (Figure 9a,b). In the 20 min coagulation time, the lowest coagulation rate was observed for PCL fiber, and the highest coagulation rate occurred with 4% PCL/aerogel fiber (Figure 8a). When the rate of aerogel in PCL was increased, the amount of blood coagulation was increased. In the 40 min coagulation time, the lowest coagulation rate was observed for 0.5% PCL/aerogel fiber, and the highest coagulation rate occurred with 4% PCL/aerogel fiber (Figure 8b). When the coagulation amounts were compared according to the coagulation times, the coagulation amounts of all fibers were decreased (Figure 9c).

### 3.4. Zone Inhibition Antimicrobial Activity Test

**Figure 10:**
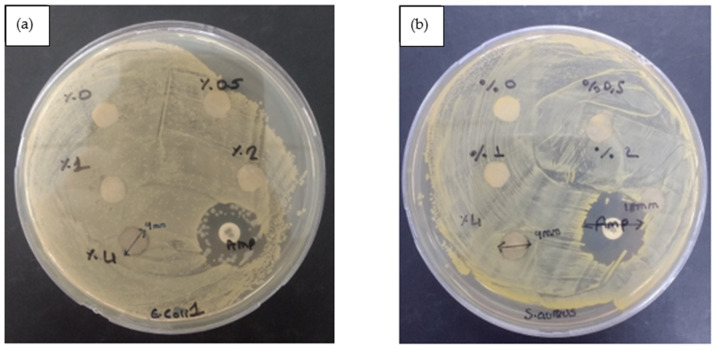
Results of zone inhibition antimicrobial activity test. 4% PCL/aerogel fibers impregnated with 0.5, 1, 2, and 4% nano-silver solution were tested with *E. coli* (**a**) and *S. aureus* bacteria (**b**).

4% PCL/aerogel fibers impregnated with 0.5, 1, 2, and 4% nano-silver solution were tested with *E. coli* and *S. aureus* bacteria, as shown in Figure 10. As a result of the test, no zone formation was observed in fibers impregnated with 0.5, 1, or 2% nano-silver solution. In addition, zone formation was observed for 4% nano-silver solution-impregnated fibers in both bacterial species. The zone inhibition of 4% PCL/aerogel fibers impregnated with 4% nano-silver solution was measured as 9 mm in *E. coli* and *S. aureus*.

## 4. Discussion

Different amounts of blood and coagulation times were used to evaluate the coagulation effect. It was observed that when the blood amount was variable, the amount of coagulation increased in the fibers. When the amount of blood decreased twofold, a 3.27-fold change was observed for the cotton surface, and a 5.66-fold change was observed for 4% PCL/aerogel fiber. When the coagulation efficiencies were compared with the amount of two times decreased blood, it was observed that the coagulation efficiency of the cotton surface and the 4% PCL/aerogel fiber was increased by 1.63 and 2.88 times, respectively. As a result, it is seen that the designed 4% PCL/aerogel fiber provides a better environment for DNA analysis and cell adhesion compared with that of the cotton surface. It was observed that when the coagulation time was variable, the amount of coagulation decreased in the fibers. When the coagulation time decreased twofold, a 1.93-fold change was observed for the cotton surface, and a 1.44-fold change was observed for 4% PCL/aerogel fiber. When the coagulation efficiencies were compared with the amount of two times decreased coagulation time, it was observed that the coagulation efficiency of the cotton surface and the 4% PCL/aerogel fiber was decreased by 3.86 and 2.88 times, respectively. As a result, it has been observed that the tolerance to timeouts of the designed 4% PCL/aerogel fiber is better during the transfer of biological samples.

PCL, which we used in our study, is thought to create a good environment for blood cells to adhere due to its hydrophobic properties. Anand et al. synthesized PCL nanofibers treated with curdlan sulfate and heparin. It was observed that the pure PCL surfaces exhibited a typical hydrophobic property, and the surfaces modified with curdlan sulfate and heparin exhibited more hydrophilic properties compared with those of the pure PCL surface. PCL-CURS and PCL-HEP surfaces observed much less adherent platelets than the pure PCL surfaces [36]. In addition, the material used with PCL must have an enhancing effect for coagulation. Jun-Yong et al. synthesized PCL nanofibers containing CaCO_3_ and β-chitosan. β-chitosan exhibits higher hydrophobicity compared with that of polycaprolactone and increases the contact area for cells to nanofibers. Calcium ions help convert prothrombin to thrombin, accelerating the formation of blood clots and catalyzing many other coagulation-related reactions. It was observed that the highest coagulation rate was observed in polycaprolactone nanofibers with CaCO_3_ content coated with β-chitosan [39].

In our study, it is thought that aerogel provides a good environment for coagulation. Jessica et. al., investigated the potential of proanthocyanidins (PA)-loaded aerogels, with their in vivo hemostatic efficacy being compared with that of an unloaded aerogel. In this assay, the values of hemostatic time and the amount of blood loss were recorded for each test material. PA-loaded aerogel and unloaded aerogel shortened the hemostatic time by more than 50% compared with other test materials and reduced the amounts of blood loss by 44% compared with the control gauze. On the other side, both aerogels achieved faster hemostasis than the other test materials, significantly reducing hemostatic time, indicating the superior hemostatic efficacy of our aerogels in controlling profuse bleeding. In the in vitro assay, whole human blood was directly added to the aerogel samples. Based on coagulation activity measurements in vitro, PAs alone showed no noticeable effects on the promotion of hemostasis. However, their inclusion into aerogel matrices favored the in vitro hemostatic activity of these composites [45]. Ning et. al., synthesized sodium alginate that was covalently modified by photosensitizers and phenylboronic acid, and cross-linked by Ca(II) ions to generate SA@TPAPP@PBA aerogel after lyophilization as an antibacterial photodynamic wound dressing. A blood cell adhesion test was performed by dropping the whole blood onto gauze, SA, SA@TPAPP, and SA@TPAPP@PBA aerogels, respectively, and incubating for 5 min at 37 °C. No obvious blood cell adhesion was observed on gauze and the other aerogel materials increased the number of adhered blood cells. Hemolysis, MTT assays, and live/dead cell staining were performed to assess the biocompatibility of SA@TPAPP@PBA aerogel. Notably, all three types of aerogels showed negligible cytotoxicity [46].

It is thought that the silica aerogel used with PCL supports the coagulation of blood cells with its high hydrophobicity. The FT-IR data we observed in our results showed that the silica aerogel-containing nanofibers have a hydrophobic structure compared to the study data of Wang et al. The hydrophobic structure of silica aerogel was observed intense spectrum in the range of 2950–2940 cm^−1^ and no observed spectrum at 1630 cm^−1^, which is the characteristic peak of the Si-OH bond [44]. 

In addition, it is thought that the fiber structure we used in our study creates an ideal environment for the adhesion of blood cells. Victoria and Ketul used PCL nanofibers treated with collagen and a different nanosurface called nanowire. The nanowire structure was fabricated using the nanotemplating technique with aluminum oxide membranes. It has been observed that the pure PCL surfaces are more hydrophobic than the other nanofiber surfaces, and the nanowire structure reduces the hydrophobicity in the synthesized nanosurfaces. The results show that nanowire surfaces reduce coagulation effects compared to other surfaces used in the study. [36].

## 5. Conclusions

In this study, polycaprolactone and aerogel biopolymers were synthesized using the electrospinning method and put a different complexion on diverse medical applications where rapid blood coagulation is required. Substances allowing the formation and stabilization of blood clots are essential in effective wound dressing. In addition, the coagulation differences between fibers and the cotton surface were revealed by blood contact analysis. It is thought that the designed surface will provide high cell adhesion with a small amount of blood. It will be the basis of different methods with increased efficiency with nanotechnological processes. It is also thought to provide a significant advantage to recover very small amounts of contaminated and undamaged DNA in biological applications. In addition, it is predicted that the designed surface can be used as an interface in nanostructured methods such as fluorescent, electrochemical, microgravimetric, enzymatic, and electroluminescence, which has recently been the most preferred ineffective and rapid DNA analysis using the AgNPs and silica.

## Figures and Tables

**Table 1:** Results of atomic and weight values of elements in fibers and mean value of fiber diameter.

	Element						Mean Fiber Diameter (nm)
	% Weight			% Atomic			
	C K	O K	Si K	C K	O K	Si K	
PCL fiber	69.41	30.59	0	75.14	24.86	0	548.226
0.5% PCL/aerogel fiber	68.17	31.64	0.18	74.10	25.82	0.09	697.553
1% PCL/aerogel fiber	68.70	30.93	0.38	74.61	25.22	0.18	616.458
2% PCL/aerogel fiber	74.75	24.54	0.71	79.96	19.71	0.32	486.598
4% PCL/aerogel fiber	67.80	31.06	1.14	74.01	25.46	0.53	523.128
Silica beads in 4% PCL/aerogel fiber	53.03	32.94	14.03	63.31	29.52	7.17	-

## Data Availability

The data presented in this study are available on request from the corresponding author.
